# Thoracoscopic surgery for congenital diaphragmatic hernia in neonates: Should it be the first choice?

**DOI:** 10.3389/fped.2022.1020062

**Published:** 2022-10-31

**Authors:** Rui Liu, Zebing Zheng, Chengyan Tang, Kaizhi Zhang, Qing Du, Yuan Gong, Daiwei Zhu, Xingrong Xia, Wankang Zhou, Lu Huang, Yuanmei Liu, Zhu Jin

**Affiliations:** ^1^ Department of Pediatric Surgery, Affiliated Hospital of Zunyi Medical University, Zunyi, China; ^2^ Department of Pediatric Surgery, Guizhou Children‘s Hospital, Zunyi, China

**Keywords:** congenital diaphragmatic hernia, neonates, thoracoscopic surgery, open surgery, repair

## Abstract

**Objective:**

Congenital diaphragmatic hernia (CDH) is an uncommon but potentially life-threatening surgical condition in neonates. Surgery can be performed by either open or thoracoscopic techniques. In this study, we compared the clinical efficacy, safety, and effectiveness of thoracoscopic and open CDH repair.

**Methods:**

A retrospective review of neonates with CDH who underwent operations at our hospital from 2013 to 2021 was performed. The various perioperative parameters were compared between neonates undergoing thoracoscopic and open surgery.

**Results:**

There were 50 neonates in this study (37 in the thoracoscopic group and 13 in the open group). Thoracoscopic surgery was associated with significantly shorter hospital stay (13.32 vs. 18.77 days, *p *< 0.001); shorter duration of postoperative mechanical ventilation (3.70 vs. 5.98 days, *p *< 0.001); early feeding (4.34 vs. 7.46 days, *p *< 0.001); and shorter time to reach optimal feeding (8.21 vs. 13.38 days, *p *< 0.001). There was one postoperative death in the open group and no death in the thoracoscopic group. The median follow-up time of the two groups was 23.8 months (20.5 months in open group and 25.0 months in thoracoscopic group). Thoracoscopic surgery was associated with lower recurrence rates, but the difference was not statistically significant (2.7% vs. 7.7%, *p *= 0.456).

**Conclusion:**

Thoracoscopy CDH repair, a safe and effective surgical technique for neonates, has better cosmesis, faster postoperative recovery, and a lower recurrence rate than other procedures. It can be considered the first choice for CDH treatment for neonates among experienced surgeons.

## Introduction

Congenital diaphragmatic hernia (CDH) is a structural defect in the diaphragm due to the lack of closure of the pleuroperitoneal folds of the embryo within 4–10 weeks after fertilization. The defect can be unilateral or bilateral and partial or complete. Due to higher intra-abdominal pressure, the abdominal organs (such as the liver, spleen, and intestine) enter the thoracic cavity through the defect and compress the adjoining lung parenchyma. CDH is a potentially life-threatening condition in neonates, with an incidence of 2–4/10,000 births (1–4).

The left-sided CDH is more common. It may be right-sided or bilateral but that is extremely rare. Diaphragmatic hernia can have varying degrees of impact on the functions of the cardiorespiratory system, gastrointestinal tract, neurocognition, and skeletal muscles (5). In right-sided CDH, the liver is usually the only prominent abdominal organ to herniate and has an echo similar to that of the lungs. Therefore, right-sided CDH is likelier to be missed. Herniation of the abdominal contents into the thoracic cavity interferes with lung development in neonates, leading to postnatal dysplasia of the lungs with reduction of pulmonary surfactants, which can lead to life-threatening pulmonary hypertension in severe cases (6, 7).

At present, surgery is the only curative treatment for CDH. Surgery can be performed by either the open or thoracoscopic techniques. However, there is still a lack of consensus on the preferred choice between these two surgical methods. In recent years, thoracoscopic surgery has been found to have several advantages over open surgery in adults. However, thoracoscopic repair for CDH in neonates is increasingly being adopted by pediatric surgeons (8). Therefore, we retrospectively compared the perioperative outcomes of thoracoscopic and open surgery for CDH in neonates to determine the preferred surgical method.

## Materials and methods

A retrospective analysis of neonates with CDH who received surgical treatment in our hospital from June 2013 to June 2021 was performed. Neonates aged less than or equal to 28 days with a confirmed diagnosis of CDH and who received primary surgical treatment at our center during the study period were included in this study. Neonates who did not receive surgery after admission or those with incomplete medical records were excluded from this study. This study was approved by our institutional ethics committee (ethical approval number: KLL-2022-475).

### Surgical methods

Open surgery: The neonates were placed in the supine position under general anesthesia. To enter the thoracic cavity, a transverse incision of 5 cm–13 cm was made in the sixth intercostal space on the side of the diaphragmatic defect. Then, the hernial contents were reduced, and the diaphragmatic defect was exposed. The defect was closed by intermittent “8” sutures using suture material (Ethicon 2–0 non-absorbable sutures), and a thoracic drainage tube was placed. Postoperatively, the neonates were shifted to the neonatal ICU, where they received ventilator support, intravenous antibiotics, anti-inflammatory drugs, and fluid supplements. The neonates were gradually weaned off the ventilator, and enteral nutrition was started after the recovery of intestinal function.

**Figure 1:**
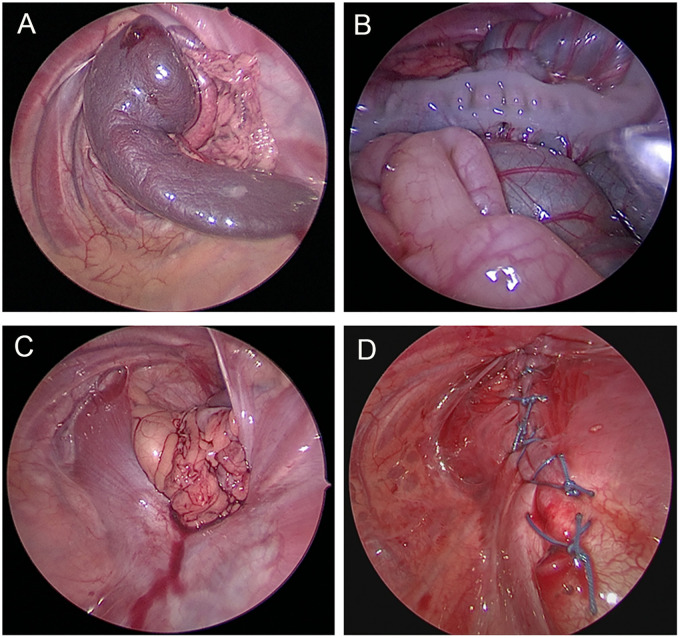
Thoracoscopic repair of congenital diaphragmatic hernia. (**A**) Herniation of the spleen into the chest cavity. (**B**) Herniation of the spleen, colon, and small intestine into the thoracic cavity. (**C**) Diaphragmatic defect after reduction of the contents. (**D**) Suture line after thoracoscopic diaphragmatic hernia repair.

Thoracoscopic surgery: The neonates received general anesthesia, and they were then placed in the lateral position with the head high and the affected side’s arm raised up to the shoulders. A 3 mm trocar was placed in the sixth intercostal space on the posterior axillary line of the affected side under direct vision. Artificial pneumothorax was created using carbon dioxide (CO_2_) with a pressure of 4–6 mmHg. Then, two 3–5 mm trocars were placed in the anterior axillary line of the sixth intercostal space and at the midpoint of the line between the subscapular angle and the spine. With the aid of pneumothorax pressure and grasping forceps, the hernial contents were gradually placed back into the abdominal cavity. Intermittent “8” sutures with 2–0 non-absorbable sutures were used to repair the diaphragm defect, and the thoracic drainage tube was inserted after the operation (Figure 1). Postoperative care was the same as that done after open surgery.

### Postoperative parameters

The following parameters were compared between the open and thoracoscopic surgery groups: duration of postoperative hospital stay, operative time, length of surgical incision, intraoperative blood loss, duration of postoperative mechanical ventilation, time to start postoperative feeding time, time to achieve target feeding time, wound infection, and recurrence rate.

### Follow-up protocol

Routine follow-up was done for 1 month, 3 months, half a year, and every year after surgery. During each visit, chest x-ray or CT scan, was performed to look for recurrence and the lung development (9).

### Statistical methods

SPSS v20.0 statistical software was used for the analysis. The measurement data were expressed as mean ± standard deviation (X ± SD). A *t*-test was used for measurement data. In some situations, the rank-sum test was used. The Chi-squared (*χ^2^*) test/Fisher’s exact test was used for categorical data. *p *< 0.05 was considered statistically significant.

## Results

### General information

A total of 53 neonates with CDH were treated during the study period. Among these, 3 cases were excluded due to incomplete treatment (*n* = 2) and incomplete medical records (*n* = 1). During the study period, open surgery was the main method between 2013 and 2016, and laparoscopic surgery was the main method between 2017 and 2021. Among these, 13 neonates underwent open surgery, and 38 cases underwent thoracoscopic surgery. One patient in the thoracoscopic group was excluded due to conversion to open surgery. No patient died in the thoracoscopic group, and one patient died during the perioperative period in the open group. In all cases, no polyester patches were used during the operations. The general characteristics of the two groups were similar, as shown in Table 1.

**Table 1:** Comparison of general characteristics of the two groups.

Group	Thoracoscopic group (*n* = 37)	Open group (*n* = 13)	t/*χ*^2^	*P*
Gender (male/female)	22/15	6/7	0.691	0.406
Gestational age	38.6 ± 1.6	38.4 ± 1.3	2.241	0.709
Birth weight (kg)	3.03 ± 0.41	3.10 ± 0.25	2.383	0.625
Side of defect (left/right)	32/5	10/3	0.655	0.413
Age at the time of surgery (day)	4.62 ± 2.52	4.56 ± 1.95	2.174	0.937
APGAR score at 1 min	5.43 ± 1.85	5.62 ± 1.94	0.303	0.763
APGAR score at 5 min	8.43 ± 1.26	8.85 ± 1.14	1.042	0.303

### Comparison of intraoperative parameters

The hernial defect size and intraoperative PaCO_2_ were similar in the two groups. The operative time and length of surgical incision in the thoracoscopic group were significantly shorter than in the open group (Table 2). Moreover, intraoperative blood loss was significantly less in the thoracoscopic group (*p *< 0.001) (Table 2).

**Table 2:** Comparison of the intraoperative parameters.

Parameters	Thoracoscopic group (*n* = 37)	Open group (*n* = 13)	t	*p*
Diameter of the defect (cm)	4.32 ± 0.91	4.69 ± 0.38	9.928	0.168
Operative time (min)	102.54 ± 33.04	129.00 ± 44.23	0.183	0.028
Intraoperative blood loss (ml)	3.45 ± 2.23	11.23 ± 5.40	25.326	<0.001
Length of the surgical incision (cm)	1.30 ± 0.27	8.54 ± 2.70	703.00	<0.001
Intraoperative PaCO_2_	42.92 ± 5.36	41.38 ± 5.04	−0.901	0.372

Surgical incision length: the length of the thoracoscopic surgical incision is the sum of all incisions (cm).

### Comparison of surgical complications

The wound infection rate and recurrence rate in the thoracoscopic group was lower than that of the open group, the survival rate was higher in the thoracoscopic group compared to the open group but not statistically significant, but all not statistically significant (Table 3).

**Table 3:** Comparison of surgical complications.

Parameters	Thoracoscopic group (*n* = 37)	Open group (*n* = 13)	χ^2^	*p*
Wound infection rate (%)	0 (0)	1 (7.7%)	2.904	0.260
Recurrence rate (%)	1 (2.7%)	1 (7.7%)	0.624	0.456
Survival rate (%)	37 (100%)	12 (92.3%)	2.904	0.260

### Comparison of postoperative recovery

Duration of hospital stay, postoperative mechanical ventilation, time to start postoperative feeding, and time to reach target feeding in the thoracoscopic group were significantly less than in the open group (all *p *< 0.05, Table 4).

**Table 4:** Comparison of postoperative recovery.

Parameter	Thoracoscopic group (*n* = 37)	Open group (*n* = 13)	t	*p*
Hospital stay (day)	13.32 ± 2.15	18.77 ± 2.89	3.771	<0.001
Postoperative mechanical ventilation (day)	3.70 ± 0.77	5.98 ± 1.06	5.276	<0.001
Postoperative start feeding time (day)	4.34 ± 0.93	7.46 ± 1.45	5.161	<0.001
Time to reach the target feeding (day)	8.21 ± 1.58	13.38 ± 2.22	1.755	<0.001

## Discussion

Since the first description of minimally invasive surgery for CDH in 1995, many centers have begun to perform thoracoscopic surgery for CDH in children (10). Indeed, thoracoscopic CDH repair has been more commonly performed for low-risk patients in recent years (11, 12). However, previous studies comparing open and thoracoscopic surgery for CDH have reported conflicting results (13–15). Some surgeons believe that open surgery has the advantages of a low postoperative recurrence rate and avoiding hypercapnia. On the other hand, studies have reported that thoracoscopic surgery is associated with a shorter operative time and a faster recovery (16, 17). Hence, we conducted this study to compare the outcomes of these two surgical treatments for CDH repair at our center.

Previous studies reported that the operative time of thoracoscopic surgery was longer than that of open surgery (18). (Kiblawi et al. (2021)) reported that despite the longer duration of thoracoscopic surgery, postoperative recovery was faster (19). (Yang et al. (2005)) recorded a mean operative time of thoracoscopic surgery of 152 min (20), whereas in the study by (Qin et al. (2019)), the mean operative time of thoracoscopic surgery was about 137.2 min, which was significantly shorter than that of open surgery (21). Lishuang et al. (2018) found that the duration of thoracoscopic surgery was about 115.6 min (8), which was similar to our study. The mean duration of thoracoscopic surgery in this study was 102 min, which was significantly shorter than the duration of open surgery (129 min). (Uecker et al. (2020)) showed that with the increasing experience of surgeons, the operation time and postoperative complications were significantly reduced (22). Moreover, advancements in endoscopic equipment and improvements in thoracoscopic skills have helped to reduce the learning curve time, thereby reducing operation time.

(Tsao et al. (2011)) found that thoracoscopic CDH repair significantly reduced the length of hospital stay but had a higher recurrence rate than open surgery (7.9% vs. 2.6%) (23). Other studies also showed that the use of thoracoscopic surgery for CDH repair is appropriate, but attention should be paid to minimizing recurrence (24, 25). Higher recurrence with thoracoscopic CDH repair may be related to the small operating space, the need for advanced surgical skills, and the steep learning curve. However, in recent years, studies have shown that with the continuous development of endoscopic technology, the recurrence rate is gradually decreasing (to even lower than that of open surgery).

A meta-analysis by (Weaver et al. (2016)) showed that infants with severe systemic diseases have a higher risk of recurrence, regardless of the surgical technique used to repair CDH (26). Studies by Al-Iede and others showed that appropriate case selection for thoracoscopy CDH repair and experienced thoracoscopic surgeons may reduce the recurrence rate of CDH (27). (De Bie et al. (2020)) and (Tyson et al. (2017)) pointed out that there was no significant difference in the recurrence rate and fatality rate between thoracoscopic and open CDH repair in neonates (28, 29). Our data showed that the recurrence rate of thoracoscopic surgery was lower than that of open surgery (2.7% vs. 7.7%), although this was not statistically significant. One neonate in this study who developed recurrence after thoracoscopic surgery had a larger hernial defect (6.5 cm). Another neonate who had recurrence after open surgery also had a large defect (5 cm). Both of these patients were reoperated on by thoracoscopy, and the hernial defect was repaired with a polyester patch and no recurrence occurred during the follow-up. Therefore, we believe that patients with larger defects may be at a higher risk of recurrence after surgery. According to our experience, recurrence can be reduced by tension-free suturing of the defect using non-absorbable stitches under thoracoscopic guidance, use of patch repair or reinforcement in patients with larger hernial defects, and use of the crochet or sled needle to externally fix the edge of the diaphragm across the rib on the rib completely closing the hernia ring and thus avoiding the risk of recurrence. This method is more convenient than the use of a needle holder to suture the diaphragm intracorporeally within the thoracic cavity and saves operation time.

The survival rate is improving with the deepening understanding and the improvement of the level of CDH in recent years. Katrin Lichtsinn et al. (2022) found that A standardized clinical practice guideline to manage patients with CDH decreased ECMO utilization and improved survival to 85% (30). Lewit RA et al. (2021) pointed out overall CDH survival is about 76.5% globally (31). Previous reports (2018) found the CDH survival rate was 94.9% (8). While our research found the survival rate was 100% in thoracoscopic group and 92.3% in open group. This may have something to do with the mild nature of our patients. The severe CDH patients were gaven up before surgery and some children with severe CDH suffered from dyspnea and hypoxemia after birth and died for their illness before reaching to hospital. Therefore, the mild patients were more common in our study.

A potential complication with thoracoscopic surgery is the development of hypercapnia and acidosis, as reported by (Bishay et al. (2013)) and (Schukfeh et al. (2020)) in prospective randomized controlled studies (32, 33). A study by (Neunhoeffer et al. (2017)) showed that thoracoscopic surgery for neonates and infants may cause a decrease in local cerebral oxygen saturation. Thus, it is recommended to avoid an inflation pressure >4 mmHg during thoracoscopic surgery (34). However, an important study by (Miranda et al. (2018)) showed that when the chest cavity of young mice was exposed to CO_2_, there were no structural, neurodevelopmental, or behavioral changes in adulthood (35). (Sidler et al. (2020)) found that under lower artificial pneumothorax pressure, hypercapnia and acidosis in CDH patients were significantly reduced (36). In our study, we maintained the artificial CO_2_ pneumothorax pressure at 4–6 mmHg. The intraoperative PaCO_2_ of the thoracoscopic group was slightly higher than that of the open group, but the difference was not statistically significant. During the operation, it is necessary to coordinate closely with the anesthesiologist team and monitor the blood gases of the patient.

As a unique advantage of minimally invasive surgery, the incision of thoracoscopic surgery is significantly reduced. Several studies have shown that thoracoscopic surgery included small incisions, good cosmesis, and lower cost (17, 21, 37). In our study, the length of the surgical incision in the thoracoscopic group was significantly shorter than in the open group. The thoracoscopic surgery requires only three 3–4 mm incisions, while the open surgery requires at least 4 cm–5 cm incisions. Importantly, the smaller incision reduced postoperative pain and allowed faster recovery after surgery.

Bawazir et al. (2021) showed that mechanical ventilation time after thoracoscopic surgery was significantly reduced compared with open surgery (3 days vs. 6 days) (38). (Szavay et al. (2012)) also showed that children undergoing thoracoscopic surgery for CDH had shorter mechanical ventilation days (18). Our results also showed that the mechanical ventilation time after thoracoscopic surgery was significantly shorter than that after open surgery (3.70 days vs. 5.98 days). The smaller incision, lesser pain, and minimal interference to the chest cavity may be responsible for reduced ventilation time in the thoracoscopic group.

The early intubation allowed early resumption of breastfeeding and shortened the time to reach the target feeding, which reduced the need for parenteral nutrition and the risk of associated infections. (Criss et al. (2018)) found that the initiation of breastfeeding after thoracoscopic surgery was significantly earlier compared to open surgery (5 days vs. 12 days), with reduced time to reach the target feeding (13 days vs. 16 days), resulting in shorter hospital stay (17.5 days vs. 20 days) (39). Bawazir et al. also reported less operative time (91 vs. 174 min), reduced duration of postoperative mechanical ventilation (3 days vs. 6 days), and shorter hospital stay (10 days vs. 12.5 days) with thoracoscopic CDH repair (36). The current study also had similar findings with early initiation of breastfeeding (4.34 days vs. 7.46 days), faster achievement of target feeding (8.21 days vs. 13.38 days), and reduced hospitalization (13.32 days vs. 18.77 days) in the thoracoscopic group.

Therefore, compared with open surgery, the advantages of thoracoscopic surgery are becoming more obvious. However, this study was a single-center retrospective study with a small sample size and limited follow-up. Hence, randomized controlled trials with longer-term follow-ups are needed to verify our results. In addition, endoscopic surgery requires professional training and it has a steep learning curve. Therefore, it is difficult to promote its widespread use.

## Conclusion

In summary, thoracoscopic CDH repair is a safe and feasible technique with the advantages of a clear surgical field, less trauma, smaller incision, faster recovery, and fewer complications compared to open CDH repair. It is believed that with advancements in endoscopic technology and the accumulation of experience, thoracoscopic surgery will become the preferred treatment for CDH.

## Data Availability

The original contributions presented in the study are included in the article/Supplementary Material, further inquiries can be directed to the corresponding author/s.
