# Supplementary material for: Thoracoscopic surgery for congenital diaphragmatic hernia in neonates: Should it be the first choice?
Source: Front Pediatr. 2022 Oct 31;10:1020062. doi: 10.3389/fped.2022.1020062 (PMC9659751; doi:10.3389/fped.2022.1020062)
Supplement: Supplementary file 1 [file Table1.doc]

| Group | Thoracoscopic group (n=37) | Open group  (n=13) | t/χ^2^ | P |
| --- | --- | --- | --- | --- |
| Gender (male/female) | 22/15 | 6/7 | 0.691 | 0.406 |
| Gestational age | 38.6±1.6 | 38.4±1.3 | 2.241 | 0.709 |
| Birth weight (kg) | 3.03±0.41 | 3.10±0.25 | 2.383 | 0.625 |
| Side of defect (left/right) | 32/5 | 10/3 | 0.655 | 0.413 |
| Age at the time of surgery (d) | 4.62±2.52 | 4.56±1.95 | 2.174 | 0.937 |
| APGAR score at 1 min | 5.43±1.85 | 5.62±1.94 | 0.303 | 0.763 |
| APGAR score at 5 min | 8.43±1.26 | 8.85±1.14 | 1.042 | 0.303 |

| Parameters | Thoracoscopic group (n=37) | Open group  (n=13) | t | p |
| --- | --- | --- | --- | --- |
| Diameter of the defect (cm) | 4.32±0.91 | 4.69±0.38 | 9.928 | 0.168 |
| Operative time (min) | 102.54±33.04 | 129.00±44.23 | 0.183 | 0.028 |
| Intraoperative blood loss (ml) | 3.45±2.23 | 11.23±5.40 | 25.326 | <0.001 |
| Length of the surgical incision (cm) | 1.30±0.27 | 8.54±2.70 | 703.00 | <0.001 |
| Intraoperative PaCO_2_ | 42.92±5.36 | 41.38±5.04 | -0.901 | 0.372 |

| Parameters | Thoracoscopic group (n=37) | Open group (n=13) | χ^2^ | p |
| --- | --- | --- | --- | --- |
| Wound infection rate (%) | 0 (0) | 1 (7.7%) | 2.904 | 0.260 |
| Recurrence rate (%) | 1 (2.7%) | 1 (7.7%) | 0.624 | 0.456 |
| Survival rate (%) | 37 (100%) | 12 (92.3%) | 2.904 | 0.260 |

| Parameter | Thoracoscopic group (n=37) | Open group (n=13) | t | p |
| --- | --- | --- | --- | --- |
| Hospital stay (d) | 13.32±2.15 | 18.77±2.89 | 3.771 | <0.001 |
| Postoperative mechanical ventilation (d) | 3.70±0.77 | 5.98±1.06 | 5.276 | <0.001 |
| Postoperative start feeding time (d) | 4.34±0.93 | 7.46±1.45 | 5.161 | <0.001 |
| Time to reach the target feeding (d) | 8.21±1.58 | 13.38±2.22 | 1.755 | <0.001 |
